# Radiomics Features on Magnetic Resonance Images Can Predict C5aR1 Expression Levels and Prognosis in High-Grade Glioma

**DOI:** 10.3390/cancers15184661

**Published:** 2023-09-21

**Authors:** Zijun Wu, Yuan Yang, Yunfei Zha

**Affiliations:** Department of Radiology, Renmin Hospital of Wuhan University, Wuhan 430000, China; zijun_wu@whu.edu.cn (Z.W.); yangyuan5244@163.com (Y.Y.)

**Keywords:** C5aR1, high-grade glioma, MRI, radiomics, prognosis, biomarker

## Abstract

**Simple Summary:**

High-grade glioma is a complex disease characterized by genome instability caused by the accumulation of genetic alterations. Identifying and evaluating the oncogenes involved is crucial for determining treatment strategies and evaluating prognosis. In this study, we suggest a potential role for C5aR1 as a biomarker of glioma prognosis. Using machine learning approaches based on paired MRI and RNA sequencing data, our results show that radiomics MRI features can be used to build models that can noninvasively predict C5aR1 expression and the prognosis of patients with high-grade glioma. The radiomics models yield satisfactory performances in predicting C5aR1 expression. In addition, our findings revealed associations between MRI radiomics and immune-related features. As an effective and reproducible tool, our radiomics model may support clinical decision making and individualized treatment.

**Abstract:**

Background: The complement component C5a receptor 1 (C5aR1) regulates cancer immunity. This retrospective study aimed to assess its prognostic value in high-grade glioma (HGG) and predict C5aR1 expression using a radiomics approach. Methods: Among 298 patients with HGG, 182 with MRI data were randomly divided into training and test groups for radiomics analysis. We examined the association between C5aR1 expression and prognosis through Kaplan–Meier and Cox regression analyses. We used maximum relevance–minimum redundancy and recursive feature elimination algorithms for radiomics feature selection. We then built a support vector machine (SVM) and a logistic regression model, investigating their performances using receiver operating characteristic, calibration curves, and decision curves. Results: C5aR1 expression was elevated in HGG and was an independent prognostic factor (hazard ratio = 3.984, 95% CI: 2.834–5.607). Both models presented with >0.8 area under the curve values in the training and test datasets, indicating efficient discriminatory ability, with SVM performing marginally better. The radiomics score calculated using the SVM model correlated significantly with overall survival (*p* < 0.01). Conclusions: Our results highlight C5aR1’s role in HGG development and prognosis, supporting its potential as a prognostic biomarker. Our radiomics model can noninvasively and effectively predict C5aR1 expression and patient prognosis in HGG.

## 1. Introduction

High-grade gliomas (HGGs) are the most common malignant brain tumors. The standard treatment guidelines recommend surgery, radiotherapy, concomitant temozolomide chemotherapy and adjuvant chemotherapy [1,2]. Despite the increased rates of maximal safe resection and considerable efforts in combination with targeted therapy, HGG progresses or inevitably recurs due to its invasive nature and treatment resistance [3]. HGG is characterized by prominent genetic heterogeneity; thus, its treatment relies on precise genetic information for its diagnostic and prognostic value [4]. Several markers, including isocitrate dehydrogenase (IDH) mutations and 1p/19q codeletion status, have become clinically used prognostic factors [5]. The integration of molecular profiling and precision therapy is important for expanding the possibilities of an effective personalized therapy for HGG.

As a recognized G-protein-coupled receptor, complement C5a receptor 1 (C5aR1) is a crucial component of the complement cascade in innate immune responses. Therapeutic C5aR1 antagonists play well-documented roles in multiple inflammatory disorders [6], and accumulating evidence indicates that C5a regulates cancer immunity [7]. The C5a/C5aR axis recruits myeloid-derived suppressor cells into tumor sites and mediates their capacity to suppress cytotoxic CD8 + T-cell function, thereby promoting tumor growth [8,9]. Additionally, elevated C5aR1 expression has been linked to poor survival in patients with head and neck squamous cell carcinomas [10]. Here, we highlight the function of the C5aR1 gene in HGG, which has not yet been studied.

The evaluation of intra-tumoral genetic heterogeneity is of great clinical significance. Accurate detection of C5aR1 status in clinical practice depends on tissue sampling through invasive surgery, which is inefficient and insufficient for acquiring the full genetic information of carcinoma tissue. Therefore, a non-invasive method that can reflect the entire genetic picture is urgently needed. Mounting evidence has shown that radiomics has various applications in HGG, including early diagnosis and typing [11,12], as well as the prediction of biological behavior and prognosis [13,14]. Certain important molecular markers, including IDH mutation and 1p/19q codeletion status, have been predicted accurately using MRI-based radiomics features [11,15]. However, so far, no study has predicted C5aR1 expression using a radiomics approach.

In the current work, we aimed to construct a preoperative contrast-enhanced MRI-based radiomics model for predicting C5aR1 expression in HGG. The aim in doing so was to investigate the association of radiomics prediction with the immunity and prognosis of patients with HGG. We then integrated bioinformatics analysis of a TCGA cohort in order to analyze C5aR1 expression in HGG and evaluated its association with clinicopathological data and prognosis. The molecular mechanisms underlying C5aR1 expression and its relationship with the immune microenvironment were also investigated.

## 2. Materials and Methods

### 2.1. Patients and Datasets

The transcriptional, clinical, and follow-up data of 298 patients with HGG were retrieved from the TCGA database (https://portal.gdc.cancer.gov/, accessed on 1 January 2023), while normal para-cancer samples were acquired from the GTEx database [16,17]. The corresponding medical imaging data were downloaded from TCIA (https://www.cancerimagingarchive.net/, accessed on 1 January 2023). For the normalization of the RNA sequencing (RNA-seq) data, raw data were transformed into transcripts per kilobase (TPM) values using the Toil software (https://github.com/BD2KGenomics/toil accessed on 1 January 2023) [18]. Clinical data included age, gender, grade, treatment regimen (radiotherapy or chemoradiotherapy), and the status of the O(6)-methylguanine-DNA methyltransferase (MGMT) promoter, IDH, and 1p/19q codeletion. The HGG cohort was first categorized into high- and low-C5aR1 groups according to the median expression value of C5aR1 using the R package Survminer.

### 2.2. Prognostic Analyses Based on the TCGA Cohort

The prognostic significance of the C5aR1 gene and clinicopathological factors for HGG were estimated using the survival package of R. Kaplan–Meier curves were first utilized for the survival analysis of each variable, and variations in survival probability among different groups were assessed using the log-rank test. Univariate Cox proportional hazards regression was used to determine variables that were significantly associated with overall survival (OS). Multivariate Cox regression analysis was performed to detect whether the significant variables were independent of other variables.

Subsequently, a subgroup analysis was conducted using univariate Cox regression to estimate the survival benefit of C5aR1 in different clinical subgroups. Hazard ratios (HR), 95% confidence intervals (CI), and *p* values were measured to express the Cox model results, which were visualized using the R package Forestplot. We performed time-dependent ROC curve analyses to validate the prognostic value of C5aR1 expression using the time-ROC package in R.

### 2.3. Comprehensive Analysis of Functional and Immune Characteristics Based on TCGA

The analysis of differentially expressed genes (DEGs) was performed with the R package DESeq2, while the GO and KEGG pathway enrichment analyses were conducted using the R package clusterProfiler [19]. An adjusted *p* value < 0.05, obtained using the Benjamini–Hochberg approach, and a false discovery rate > 0.1 were considered significant. Moreover, for the analysis of immune infiltration, the gene expression profiles (in TPM format) were imported into CIBERSORTx (https://cibersortx.stanford.edu/ accessed on 1 January 2023) to examine the relative fraction of 22 types of immune cells with 1000 permutations [20], and the variations in the immune cell fraction between the high- and low-C5aR1 groups were compared using the R package Limma.

### 2.4. MRI Images Preprocessing

Spatial resampling, image intensity normalization, and bias field correction were applied to reduce MR signal intensity inhomogeneity. We used the N4ITK algorithm for bias field correction to minimize the effects of magnetic field inhomogeneity.

### 2.5. Volume-of-Interest Segmentation

The 3D Slicer software (version 4.10.2) was used for manual volume-of-interest (VOI) segmentation. Each image was segmented by a senior radiologist with more than 10 years of working experience, who was blinded to the patient’s clinical data or diagnosis.

Twenty patients were randomly selected to evaluate the reliability of radiomics features. The obtained images were analyzed and segmented by an experienced radiologist. Based on the 20 paired segmentation results, radiomics features were automatically extracted, and the intraclass correlation coefficient (ICC) was calculated and utilized to measure inter- and intra-observer agreement.

### 2.6. Radiomics Features Extraction and Normalization

Radiomics features were obtained using PyRadiomics [21]. A total of 107 features were extracted in order to analyze VOI’s shape, size, intensity, morphology, and texture. The radiomic features were normalized separately for each scanner type and for each training and testing set (standardization by z-scores algorithm in the caret package of R, i.e., mean: 0 and standard deviation: 1) to minimize the effects of variability between scanner types (1.5T vs. 3T).

### 2.7. Radiomics Features Selection

Features with ICC values above 0.75 were chosen for further analysis. To prevent overfitting, we analyzed these features and removed redundant subsets using a feature selection procedure. The maximum relevance–minimum redundancy (mRMR) approach was used to rank the importance of features (“mRMR” package in R CRAN). The input features were ranked by optimizing the mutual information (MI) between class labels and minimizing the MI between other features. Features selected using the mRMR method were further screened using recursive feature elimination (RFE). Using RFE, the features were selected by gradually decreasing the number of features studied. Features with absolute minimum weight were removed from the feature set until the remaining features reached the desired number.

### 2.8. Logistic Regression and Support Vector Machine Model Establishment 

Two machine learning approaches were used to develop the radiomics prediction models for the experiment: logistic regression (LR) and support vector machine (SVM). The remaining features with nonzero coefficients were retained and used in a multivariate backward stepwise LR with the minimum Akaike information criterion to establish the model. The use of SVM as a classifier is primarily based on its ability to generate classification hyperplanes, such that the margins between them and the closest instances are low. After retaining the best-performing model, the radiomics score (Rad-score) was calculated.

### 2.9. Model Validation and Evaluation

ROC analysis was used to determine the diagnostic significance of the current models. The assessment metrics included accuracy, specificity, sensitivity, positive predictive value and negative predictive value. A calibration curve was plotted to identify the calibration of the radiomics model using the Hosmer–Lemeshow goodness-of-fit test. The Brier score assessed the overall model performance (measure package in R). The pROC package in R was used to calculate the area under the ROC curve. The R tool rmda package was used to analyze the decision curves.

### 2.10. TCIA and TCGA Data Combination

By combining the TCIA and TCGA datasets, we obtained 182 overlapping data points of patients with HGG. The Rad-score calculated using the SVM model was merged with the clinical data. Next, we used the cutoff value of the Rad-score to classify the Rad-score as a binary categorical variable, with low vs. high groups. Based on the low- and high-Rad-score group, we constructed a clinical characteristics table using the CBCgrps R package. The cutoff value was calculated using the survminer R package. The correlation between the Rad-score and immune-related genes [22] was analyzed using Spearman’s correlation (stats packages in R).

### 2.11. Statistical Analysis

The Wilcoxon rank-sum test was used to analyze differences in C5aR1 expression between HGG and para-cancer tissues. Wilcoxon rank-sum and Pearson’s χ^2^ test were performed to compare continuous and categorical clinical variables between the two groups, respectively. Spearman’s correlation was used to estimate the correlation between C5aR1 and clinicopathological variables, and the results are presented in a heatmap. Statistical tests were performed using R; in all cases, the two-sided significance level was set at 0.05. 

**Figure 1:**
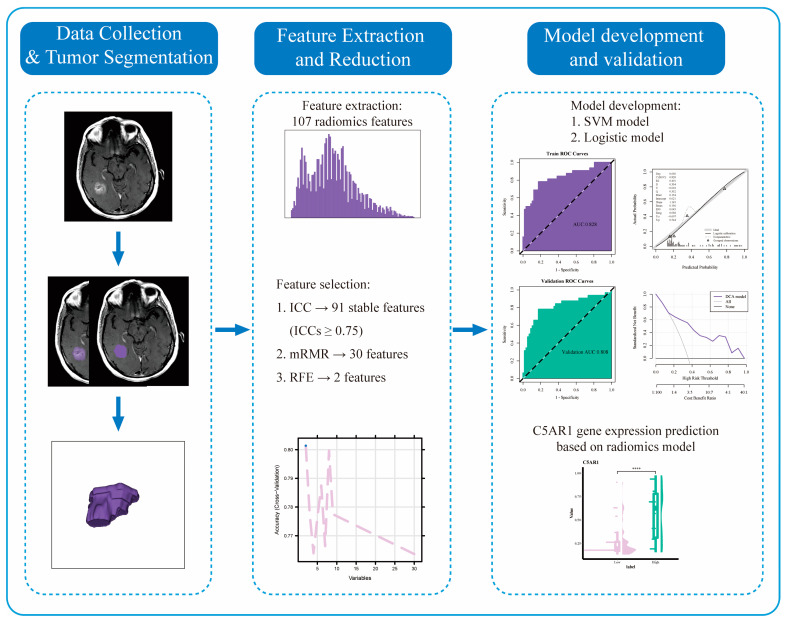
Overall workflow and pipeline of the project. ****, *p* < 0.0001.

The overall study workflow is shown in Figure 1.

## 3. Results

### 3.1. *C5aR1* Expression and Correlation with Clinical Baseline Characteristics

**Figure 2:**
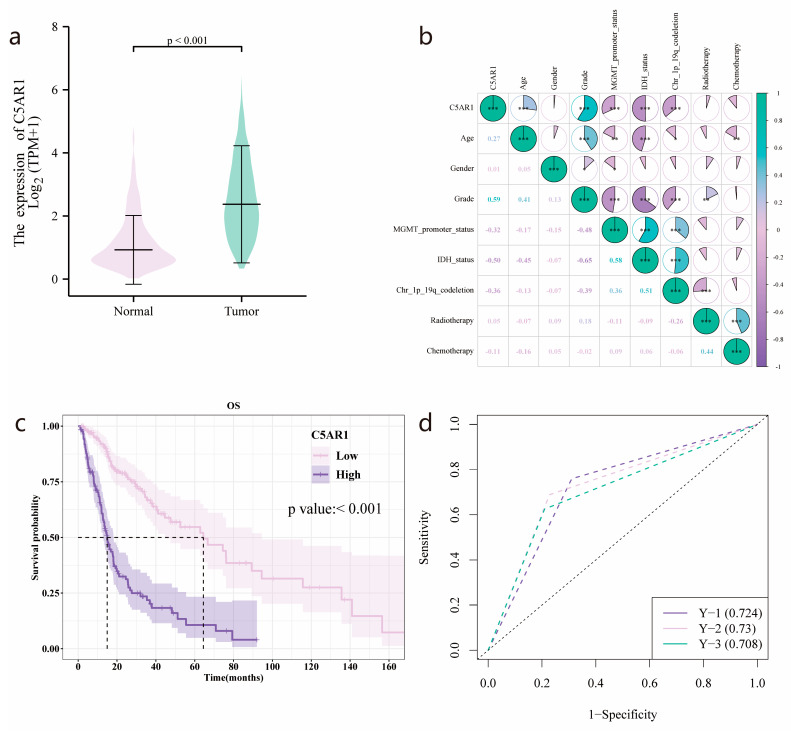
C5aR1 expression and clinical correlation in HGG according to TCGA. (**a**) Higher C5a receptor 1 (C5aR1) expression was observed in high-grade glioma (HGG) samples compared with normal tissues. (**b**) Correlation of C5aR1 expression with clinicopathologic features. (**c**) Impact of C5aR1 expression on overall survival in Kaplan–Meier curves. (**d**) Time-dependent receiver operating characteristic (ROC) analysis of C5aR1 expression for 1-, 2-, and 3-year survival prediction. *, *p* < 0.05; **, *p* < 0.01; ***, *p* < 0.001.

All 298 patients with HGG obtained from the TCGA database were categorized into two groups based on a cutoff value of 2.6834 for subsequent analyses, with one group with high (174 samples) and one with low (124 samples) C5aR1 expression. Table 1 shows significant variations observed between the high- and low-C5aR1 groups for age, grade, status of MGMT promoter, IDH, and 1q/19q codeletion, with all associated *p* values being less than 0.001. No statistically significant differences were found for gender, radiotherapy, or chemotherapy between the two groups. Wilcoxon rank-sum testing revealed that C5aR1 expression was markedly elevated in the HGG samples compared to that seen in the normal para-cancer samples (*p* < 0.001; Figure 2a).

Moreover, as shown in Figure 2b, C5aR1 expression positively correlated with age (Spearman’s *R* = 0.27) and grade (Spearman’s *R* = 0.59). A moderate negative association was observed between C5aR1 expression and MGMT promoter status (Spearman’s *R* = −0.32), IDH status (Spearman’s *R* = −0.5), and 1q/19q codeletion (Spearman’s *R* = −0.36). No correlations were found between gender, radiotherapy, or chemotherapy.

### 3.2. Survival Outcomes and Multivariate Analysis of Prognostic Factors for HGG

**Figure 3:**
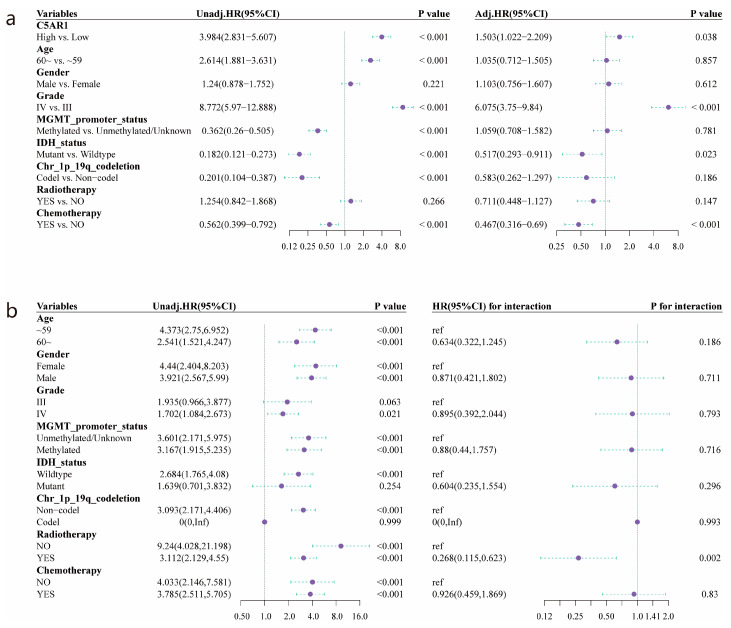
Prognostic analyses based on the TCGA cohort: (**a**) Univariate and multivariate Cox regression analysis of C5aR1 and clinicopathologic factors. (**b**) Subgroup analysis and interaction test for the prognostic value of C5aR1.

As illustrated by Kaplan–Meier survival analysis (Figure 2c), a significantly shortened OS was detected in patients with high C5aR1 expression relative to those with low C5aR1 expression (*p <* 0.001), with a median OS of 14.933 months (95% CI: 12.8–18.133) vs. 64.433 (95% CI: 44.633–94.5). Furthermore, the AUC of the time-dependent ROC curve for C5aR1 expression was 0.724, 0.73, and 0.708 at the 1-, 2-, and 3-year follow-up, respectively (Figure 2d). Univariate Cox regression analysis for OS in Figure 3a revealed that high C5aR1 levels correlated with poorer survival (HR: 3.984, 95% CI: 2.831–5.607, *p* < 0.001). In multivariate Cox regression analysis (Figure 3a), C5aR1 showed an independent prediction ability for OS (HR: 1.503, 95% CI: 1.022–2.209, *p* < 0.05), as did grade, IDH status, and chemotherapy. Conversely, age, MGMT promoter, and 1q/19q codeletion status did not.

We further investigated the effect of C5aR1 expression on prognosis using a subgroup analysis stratified by clinical traits (Figure 3b). These findings suggested that C5aR1 was a risk variable for OS among all subgroups, including age, gender, MGMT promoter status, radiotherapy, and chemotherapy (*p* < 0.001). 

### 3.3. Immune Cell Fraction Analyses and GO/KEGG Pathway Analysis for Patients with High- and Low-*C5aR1* Expression

**Figure 4:**
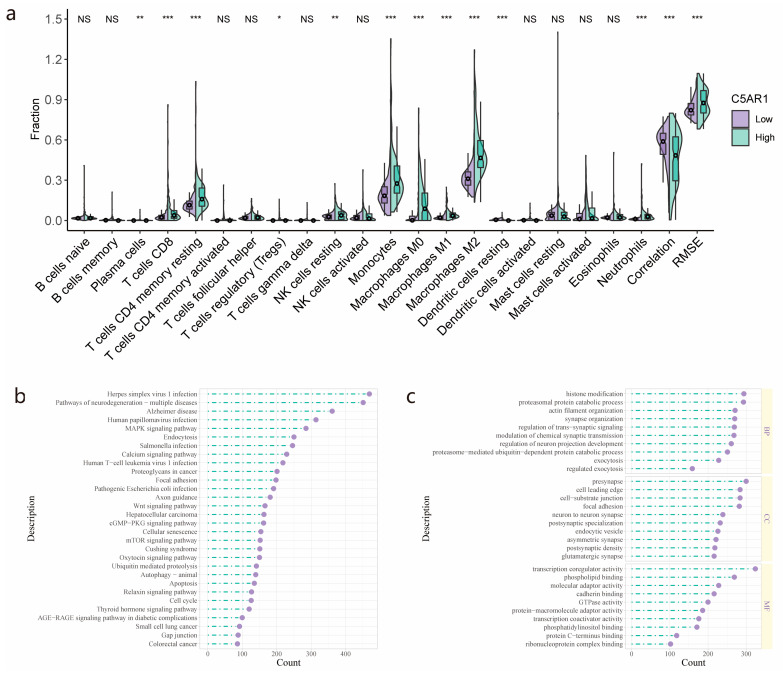
C5aR1-related immune infiltration analysis and pathway enrichment: (**a**) The differences in the fraction of 22 immune cell types between the high- and low-C5aR1 expression groups from the TCGA cohort. (**b**) Significantly enriched KEGG pathways and (**c**) GO annotations of C5aR1-related genes. NS, no significance; *, *p* < 0.05; **, *p* < 0.01; ***, *p* < 0.001.

We investigated the variation in the fraction of the 22 immune cell types between patients with high and low C5aR1 (Figure 4a). Eight types of immune cells, including T cells CD8, T cells CD4 memory resting, monocytes, and macrophages M0, M1, and M2, showed greater fractions in the high-C5aR1 group, with significant differences compared to the low-C5aR1 group (*p* < 0.001). Among immune cells, the M2 macrophages were the most abundant, especially in the high-C5aR1 group. We moreover identified the DEGs between patients with high- and low-C5aR1 expression. The 30 most significantly enriched KEGG and GO terms, following the GO and KEGG enrichment analyses of the identified DEGs, are shown in Figure 4b and Figure 4c, respectively.

### 3.4. Radiomics Features Selection

**Figure A1:**
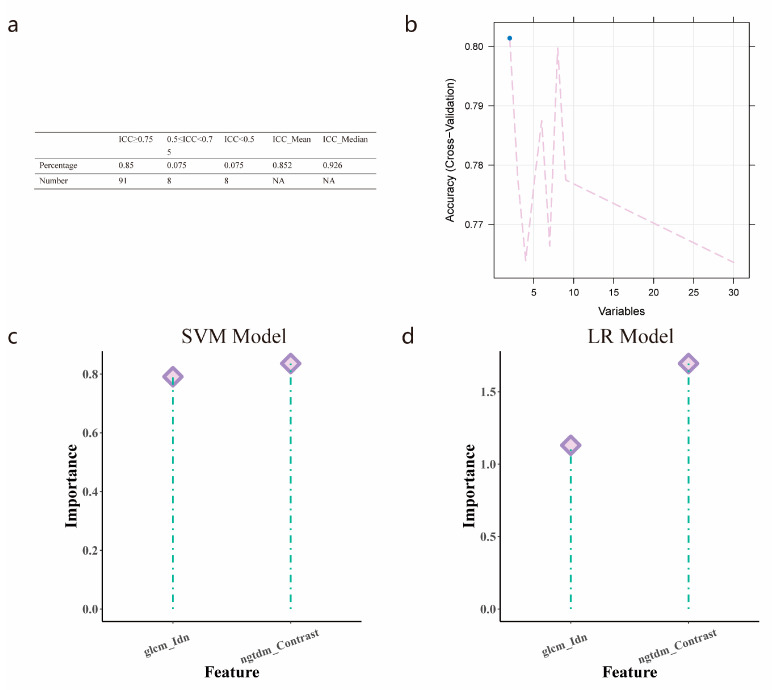
Selection of radiomics features: (**a**) The evaluation of inter- and intra-observer agreement on ROI extraction by intraclass correlation efficient. (**b**) Recursive feature elimination method to further select features. (**c**,**d**) The importance of the two optimal features to SVM and LR algorithms, respectively.

Figure A1a shows that the median ICC value of radiomics features was 0.926. There were 91 features with ICC values ≥ 0.75, accounting for 85% of all features. These features were used for further feature selection. The mRMR method was used to select the 30 highest mRMR-ranked features. The RFE method was employed to select the best feature group. Two optimal features (original_ngtdm_Contrast and original_glcm_Idn) were screened using the mRMR and RFE algorithms (Figure A1).

### 3.5. Establishment and Validation of Logistic Regression and SVM Models

**Figure 5:**
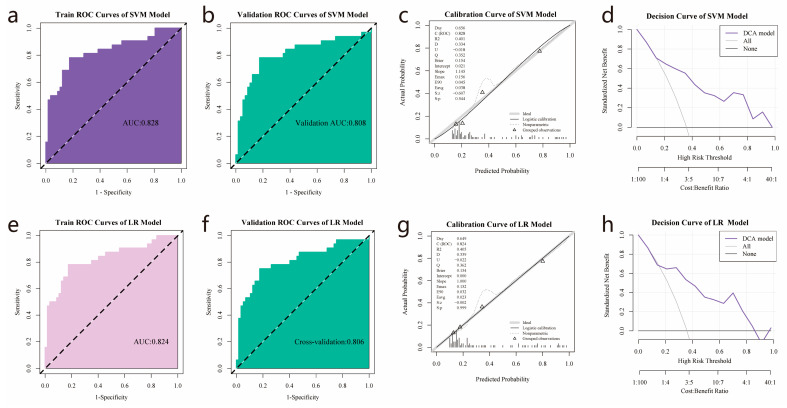
Radiomics models’ performance in predicting C5aR1 expression for 182 patients with HGG from the TCIA-TCGA intersection cohort: (**a**,**b**) ROC curves of the support vector machine (SVM) model in the training and validation cohorts, with (**c**,**d**) being the associated SVM calibration and decision curves. (**e**,**f**) The logistic regression model ROC curves in the training and validation cohorts, with (**g**,**h**) being the accompanying calibration and decision curves.

Two optimal features were selected for the LR and SVM modeling analysis. Figure 5 depicts the performance of the C5aR1 gene expression prediction models in the training and validation cohorts. The SVM model showed satisfactory predictive ability, with an AUC of 0.828 (Figure 5a), and a 10-fold cross-validation returned a similar AUC estimate (Figure 5b). The calibration curve and Hosmer–Lemeshow goodness-of-fit test indicated a relatively high degree of concordance between the SVM model’s prediction and actual observations (*p* > 0.05, Figure 5c). The decision curve analysis showed that the SVM model was clinically practical (Figure 5d).

As shown in Figure 5e, The LR model also demonstrated satisfactory predictive ability with an AUC of 0.824, and cross-validation showed an AUC of 0.806 (Figure 5f). According to the calibration curve, the LR model exhibited a considerable level of concordance with real observations in relation to the expression level of the C5aR1 gene (*p* > 0.05, Figure 5g). The LR model also had clinical practicality, as demonstrated by the decision curve analysis (Figure 5h).

The AUC values of the SVM model and the 10-fold internal cross-validation were comparable to those of the LR model (Table 2). The DeLong test was employed to confirm the comparison of ROCs, and the findings indicated a non-significant statistical difference between the two models (*p* = 0.317 in the training set and *p* = 0.982 in the validation set). Based on the above results, we used the Rad-score output from the SVM model for further clinical analyses, it being the most optimal between the two models.

### 3.6. TCIA and TCGA Data Combination

Based on the intersection of the TCIA images and the TCGA clinical data, 182 patients were included in the subsequent analysis. Using 0.2424 as the cutoff value, the patients were categorized into high (n = 119)- and low (n = 63)-Rad-score groups. As shown in Table 3, the Rad-score significantly correlated with age, WHO grade, IDH status, 1p/19q codeletion, MGMT promoter status, and OS (*p* < 0.001). No significant relationship was detected between the Rad-score and other clinicopathological characteristics.

### 3.7. Validation of the Prognostic Value of the Radiomics Score

**Figure 6:**
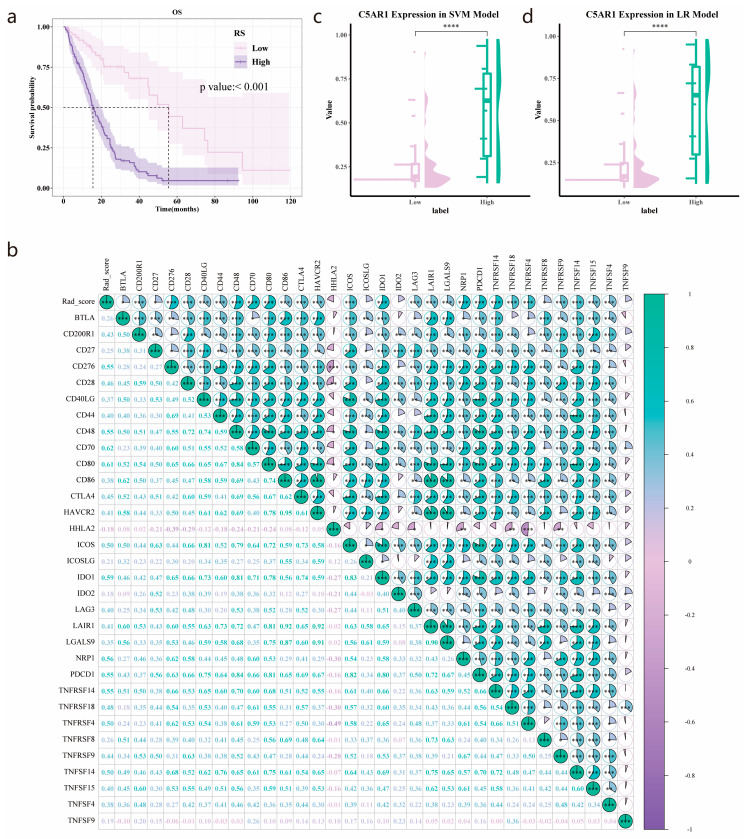
Analyses of Rad-score based on the TCIA-TCGA intersection cohort: (**a**) Kaplan–Meier curve showing the association of high radiomics (Rad)-score with worse overall survival of patients and the median survival time in the high- and low-Rad-score groups. (**b**) The correlations between Rad-score and immune-related genes. (**c**,**d**) Difference in C5aR1 expression between high- and low-Rad-score groups in the SVM and LR models, respectively. *, *p* < 0.05; **, *p* < 0.01; ***, *p* < 0.001; ****, *p* < 0.0001.

According to Kaplan–Meier curves, a high Rad-score significantly linked to poorer OS (*p* < 0.001) (Figure 6a). The median survival time was 15.57 and 55.53 months in the high- and low-Rad-score groups, respectively. We also analyzed the association between the Rad-score and immune-related genes (Figure 6b), observing that the Rad-score positively correlated with CD70 (Spearman’s *R* = 0.62, *p* < 0.001), CD80 (Spearman’s *R* = 0.61, *p* < 0.001), and IDO1 (Spearman’s *R* = 0.59, *p* < 0.001).

### 3.8. *C5aR1* Expression Analysis in the SVM and Logistic Regression Model

As shown in Figure 6c, the expression levels of C5aR1 were higher in the high Rad-score group in comparison to the low group. Similar results were also obtained for the LR model (Figure 6d).

## 4. Discussion

In this study, we first established the prognostic role of C5aR1 in patients with HGG by investigating RNA-seq data. Patients with HGG who also presented with higher C5aR1 gene expression have a worse prognosis. C5aR1 expression was associated with advanced clinicopathological features (old age and high grade), as well as with the status of MGMT promoter, IDH, and 1q/19q codeletion. We further uncovered the biological significance underlying C5aR1 expression in HGG. We then developed and validated a contrast-enhanced T_1_WI MRI-based radiomics model in order to preoperatively evaluate the expression level of C5aR1 in HGG. Our results showed that a higher Rad-score was linked to a higher C5aR1 expression and poorer survival, and additional associations were observed between the Rad-score and immune-related gene sets. The current study employed radiomic analysis of MRI images to predict the status of C5aR1 expression, which may contribute to making more precise clinical decisions.

Several studies have demonstrated the existence of an association between high C5aR1 expression and poor outcomes in head and neck squamous cell carcinoma [10], gastric cancer [23], breast cancer [24], and glioblastoma [25]. Our current work focused on the field of HGG and we observed that patients with increased C5aR1 expression had worse OS. C5aR1 was an independent prognostic factor, as determined using univariate and multivariate Cox analysis.

To understand the potential biological underpinnings of the prognostic significance of C5aR1, RNA-seq data of gliomas was used to reveal the immunological and functional nature of C5aR1. The two C5aR1 subgroups showed differential immune cell compositions, among which M2 macrophages and monocytes were more enriched in the C5aR1 high-expression phenotype. Infiltration of myelomonocytic cells, specifically macrophages and monocytes, is a common denominator in cancer [26]. The M2 macrophages have been involved in tumor progression in gliomas via an immunosuppressive effect [27,28,29]. Chen et al. discovered that bone marrow-derived monocytes/macrophages infiltrate glioblastomas early in tumorigenesis [30]. Therefore, the effect of C5aR1 on prognosis may be related to the recruitment of M2 macrophages and monocytes [31]. Through GO and KEGG analyses, we further identified that C5aR1 was associated with Alzheimer’s disease-related pathways and tumor development-related pathways, e.g., the MAPK signaling cascade. These findings provide clues regarding the function of C5aR1 in HGG development. Additional research would be warranted to more comprehensively characterize the underlying regulatory mechanisms of C5aR1 in HGG.

Radiomics features have been reported as potential markers for predicting the molecular characteristics of gliomas [32,33]. Several biomarkers, including the status of 1p/19q codeletion (AUC 0.72–0.96) [34,35] and MGMT methylation (AUC 0.88) [36], have been predicted by MRI radiomics. Here, we extracted radiomics features from contrast-enhanced T_1_WI MRI images to construct a predictive model and calculated a Rad-score to predict C5aR1 expression levels in HGG. Two radiomic texture features (original_glcm_Idn, and original_ngtdm_Contrast), which define the association between adjacent voxels, were selected to construct the model. As a commonly used trait, the gray-level co-occurrence matrix (glcm) provides second-order statistics for grayscale data. The original_glcm_Idn reflects local heterogeneity within tumors, whereas the original_neighboring gray tone difference matrix (ngtdm)_Contrast measures the variation between gray and the average gray values at adjacent distances [37]. Our model used two common classifiers, SVM and LR, that showed similar favorable performances. Our two radiomics models achieved high predictive quality in the training and validation subsets, with AUCs of 0.828 and 0.808 for the SVM model and 0.824 and 0.806 for the LR model, respectively. Based on our observations, we conclude that MRI radiomics is an effective tool for predicting C5aR1 expression.

We further explored the association between the Rad-score and OS in a radiomics cohort with paired MRI and clinical data (n = 182). MRI radiomics have been used to estimate the OS in multiple cancer types, with several results stemming from gliomas. A glioblastoma study by Choi et al. reported that patients with a high risk Rad-score had a shorter OS [38]. Li et al. developed a 14-feature MRI radiomics model that showed the prognostic significance and predictive power of OS in glioma [13]. Two research teams indicated that the performance of radiomics was superior to that of clinical parameters, exploring the possibility of utilizing radiomic features to anticipate progression-free survival [39,40]. In our present work, a high Rad-score was significantly associated with poor prognosis in patients with HGG, which is consistent with previous reports. Altogether, the radiomics model showed great potential for risk stratification based on patient OS.

However, our approach had several limitations. First, the retrospective nature of the study meant that the influence of confounding factors was inevitable. Moreover, this was a single-center study with a small sample size, and the findings required additional verification by large-sample multicenter studies. Third, the patients included herein were recruited from public datasets and data heterogeneity may have been present. In planned follow-up investigations, we will collect our own data to further verify the predictive reliability of our developed models.

## 5. Conclusions

The expression level of C5aR1 was significantly linked to the prognosis of patients with HGG. Radiomics features based on contrast-enhanced T_1_WI MRI were linked to prognosis in patients with HGG, highlighting their potential as a robust noninvasive solution for predictively assessing C5aR1 expression in such patients. Our predictive model may be used as an effective, reproducible, and generalizable tool for the noninvasive characterization of gliomas in order to support individualized treatment.

## Figures and Tables

**Table 1:** Clinical characteristics of patients with glioma from TCGA.

Variables	Total (n = 298)	Low Expression of C5aR1 (n = 174)	High Expression of C5aR1 (n = 124)	*p*
Age, n (%)				<0.001
~59	197 (66)	134 (77)	63 (51)	
60~	101 (34)	40 (23)	61 (49)	
Gender, n (%)				0.917
Female	120 (40)	71 (41)	49 (40)	
Male	178 (60)	103 (59)	75 (60)	
Grade, n (%)				<0.001
III	170 (57)	142 (82)	28 (23)	
IV	128 (43)	32 (18)	96 (77)	
MGMT_promoter_status, n (%)				<0.001
Unmethylated/Unknown	118 (40)	46 (26)	72 (58)	
Methylated	180 (60)	128 (74)	52 (42)	
IDH_status, n (%)				<0.001
Wildtype	169 (57)	62 (36)	107 (86)	
Mutant	129 (43)	112 (64)	17 (14)	
Chr_1p_19q_codeletion, n (%)				<0.001
Non-codel	248 (83)	125 (72)	123 (99)	
Codel	50 (17)	49 (28)	1 (1)	
Radiotherapy, n (%)				0.466
NO	70 (23)	44 (25)	26 (21)	
YES	228 (77)	130 (75)	98 (79)	
Chemotherapy, n (%)				0.078
NO	79 (27)	39 (22)	40 (32)	
YES	219 (73)	135 (78)	84 (68)	

**Table 2:** The performance of the SVM and LR models.

	Training Cohort					Validation Cohort				
	AUC (95% CI)	ACC	SPE	SEN	*p*	AUC (95% CI)	ACC	SPE	SEN	*p*
SVM	0.828 (0.731–0.924)	0.809	0.825	0.781		0.808 (0.703–0.913)	0.809	0.825	0.781	
LR	0.824 (0.727–0.921)	0.809	0.825	0.781		0.806 (0.703–0.91)	0.798	0.825	0.75	
SVM vs. LR					0.317					0.806

AUC, area under the curve; SVM, support vector machine; LR, logistic regression; ACC, accuracy; SPE, specificity; SEN, sensitivity.

**Table 3:** Clinical characteristics of patients with glioma from combination of TCIA and TCGA.

Variables	Total (n = 182)	Low Radiomics Score (n = 63)	High Radiomics Score (n = 119)	*p*
Gender, n (%)				0.033
Female	80 (44)	35 (56)	45 (38)	
Male	102 (56)	28 (44)	74 (62)	
Age, n (%)				<0.001
~59	112 (62)	53 (84)	59 (50)	
~60	70 (38)	10 (16)	60 (50)	
Grade, n (%)				<0.001
III	62 (34)	51 (81)	11 (9)	
IV	120 (66)	12 (19)	108 (91)	
MGMT_promoter_status, n (%)				<0.001
Unmethylated/Unknown	94 (52)	19 (30)	75 (63)	
Methylated	88 (48)	44 (70)	44 (37)	
Chemotherapy, n (%)				0.129
NO	39 (21)	9 (14)	30 (25)	
YES	143 (79)	54 (86)	89 (75)	
Radiotherapy, n (%)				0.059
NO	34 (19)	17 (27)	17 (14)	
YES	148 (81)	46 (73)	102 (86)	
IDH_status, n (%)				<0.001
Wildtype	134 (74)	24 (38)	110 (92)	
Mutant	48 (26)	39 (62)	9 (8)	
Chr_1p_19q_codeletion, n (%)				<0.001
Non-codel	163 (90)	46 (73)	117 (98)	
Codel	19 (10)	17 (27)	2 (2)	
OS, n (%)				<0.001
0	57 (31)	40 (63)	17 (14)	
1	125 (69)	23 (37)	102 (86)	
OS time, Median (Q1, Q3)	17.02 (9, 27.55)	21.27 (13.85, 38.07)	14.93 (7.57, 24.57)	<0.001

OS, overall survival.

## Data Availability

Publicly available datasets were analyzed in this study. This data can be found in: TCGA (portal.gdc.cancer.gov/, accessed on 1 January 2023); GTEx (https://gtexportal.org/home, accessed on 1 January 2023); and TCIA (https://tcia.at/home, accessed on 1 January 2023).
